# Long-term high physical activity modulates event-related potential indices of inhibitory control in postmenopausal women

**DOI:** 10.7717/peerj.6523

**Published:** 2019-03-18

**Authors:** Chang Xu, Yingzhi Lu, Biye Wang, Chenglin Zhou

**Affiliations:** 1 School of Psychology, Shanghai University of Sport, Shanghai, China; 2 College of Physical Education, Yangzhou University, Yangzhou, China

**Keywords:** Physical activity, Event-related potential, Inhibition control, Postmenopausal women

## Abstract

**Background:**

Inhibition processing is sensitive to aging, and an age-related decline in inhibition processing has been associated with an accelerated rate of progression to Alzheimer disease. Elderly women are two to three times more likely than age-matched men to have Alzheimer disease. Therefore, this study examined whether long-term high physical activity affects inhibitory processing, specifically among postmenopausal women.

**Methods:**

In total, 251 candidates were screened using the Montreal Cognitive Assessment and the Raven’s Standard Progressive Matrices to assess their cognitive abilities and the International Physical Activity Questionnaire (Chinese version) to assess their physical activity levels. The participants were then grouped into either a long-term high physical activity group (defined as more than 3 days of high intensity activity per week and gross metabolic equivalent minutes (MET-minutes) higher than 1,500 MET-minutes/week or a gross MET higher than 3,000 MET-minutes/week obtained through walking or other moderate or high intensity activity) or a control group and matched for demographic and health characteristics as well as cognitive scores. Event-related potentials (ERPs) were recorded as participants performed a Go/No-go task to assess inhibition processing.

**Results:**

The long-term high physical activity group (*n* = 30) had faster Go reaction times than the control group (*n* = 30), whereas no significant difference between the two groups was found in their performance accuracy on the No-go task. For the ERP results, the latency of N2 component was significantly shorter in the long-term high physical activity group than that in the control group.

**Discussion:**

The results of this study suggested that long-term high physical activity may increase the efficiency of the inhibitory control system by increasing the activity of response monitoring processes.

## Introduction

Previous research has revealed that inhibition processing shows a longitudinal decline with aging, suggesting that this ability is particularly sensitive to age-related declines among biological processes (Goh, An & Resnick, 2012). Although the menopause is a nature processing for aged women, it is not hard to understand the influence on hormone level that the menopause would bring with. For example, the decreased estradiol and progesterone, which play an important role with the inhibitory control, not only influence the homeostasis, but also negative to the cognitive ability, such as the memory, emotion regulation, execution control, and so on (Barth, Villringer & Sacher, 2015). In this way, lacking these kinds of hormones, the postmenopausal women were revealed showing a higher risk than elderly men to have Alzheimer disease (Petersen, 2004). Thus, considering this high risk of elderly females compared with the males, research on inhibitory processing in aging adults, especially women, is critically important to public health.

Recently, physical activity is more and more popular in the promotion of public health. Physical activity is defined as any bodily movement produced by skeletal muscles that result in energy expenditure (Caspersen, Powell & Christenson, 1985). Considering the myriad potential benefits of exercise and fitness, increasing physical activity has been suggested for aging adults (Chang et al., 2012). For example, it has been shown that for older adults without known cognitive impairment, long-term high physical activity can improve their cognitive functions, including inhibitory control (Angevaren et al., 2008). In addition, physical activity also reduced activation of the anterior cingulate cortex, a brain region associated with inhibitory control (Aron & Poldrack, 2005). On the other hand, after assessments of cognitive performance with 18,766 US aged women, Weuve et al. (2004) found a 20% lower risk of cognitive impairment for women in the highest quintile of activity. Another study obtained 5,925 aged women’s cognitive performance by questionnaires, and concluded that women with higher levels of baseline physical activity were less likely to develop cognitive decline (Yaffe et al., 2001). These results promoted that the physical activity would improve aged people, including aged women’s inhibitory control ability. Usually, most of the aged women are postmenopausal individuals, so previous studies did not take the menopause as a specific factor or a recruit condition. However, the menopause would cause a series of impact on both physical and mental. Such, it is necessary to consider the menopause as one of the recruit conditions, and to investigate the relationship between physical activity and inhibitory control in postmenopausal women only.

To assess inhibitory control, many previous studies have examined event-related potentials (ERPs) while animals or humans performed Go/No-go tasks (Falkenstein, Hoormann & Hohnsbein, 1999; Bokura, Yamaguchi & Kobayashi, 2001). We used this same paradigm in the present study. In the Go/No-go paradigm, participants respond to a given target stimulus in Go trials but must withhold a response to the target stimulus in No-go trials. Two major ERP components have been consistently linked with inhibitory control processes. The N2 component, which is a negative-going wave elicited in the frontal region of the brain that peaks within 260 ms, is thought to represent conflict monitoring processes (Folstein & Van Petten, 2008) and to be related to upstream processes involved in response inhibition or conflict processing (Polich & Kok, 1995). While, in Go/No-go task, it is common to observe a larger frontal N2 and frontocentral P3 on trials where inhibition is needed (Smith, Johnstone & Barry, 2006, 2008).

Thus, the aim of the present study was to investigate whether long-term high physical activity specifically affects inhibitory control among aged women by assessing the neurophysiological correlates of inhibitory control using ERPs. To this end, individuals who had engaged in long-term high physical activity were assigned to one group, and those whose physical activity was within the normal range were assigned to a control group. We recorded high-resolution ERPs in the individuals of both groups during their performance on a Go/No-go task. On the basis of the results of previous studies examining physical activity and cognitive performance among aged women, we hypothesized that the postmenopausal women with higher physical activity would perform better on the No-go trials, which require inhibitory control, showing greater N2 and P3 amplitudes than the women in the control group owing to better inhibitory control ability. Also, the faster reaction time could be found in the Go trials, among the higher physical activity individuals, and the shorter N2 and P3 latency as well.

## Methods

### Ethical approval

The study was carried out ethically and approved by the Ethical Committee of Shanghai University of Sport (No. 2015024).

### Participants

All participants were recruited from healthy women in Shanghai, China, through advertisements and lectures placed in local community health centers. All were right-hand dominant and had normal or corrected to normal vision. Participants all signed informed consent forms and were told the purpose of the study.

The eligibility criteria included (1) having more than 12 years of education, (2) being between 55 and 65 years old, and (3) being menopausal for at least 12 months. The exclusion criteria included (1) a diagnosis of any metabolic disease, such as diabetes, (2) a diagnos is of cardiovascular disease, such as coronary heart disease, (3) a diagnos is of mental disorder, such as depression, or (4) a family history of an autosomal dominant hereditary disease.

**Figure 1:**
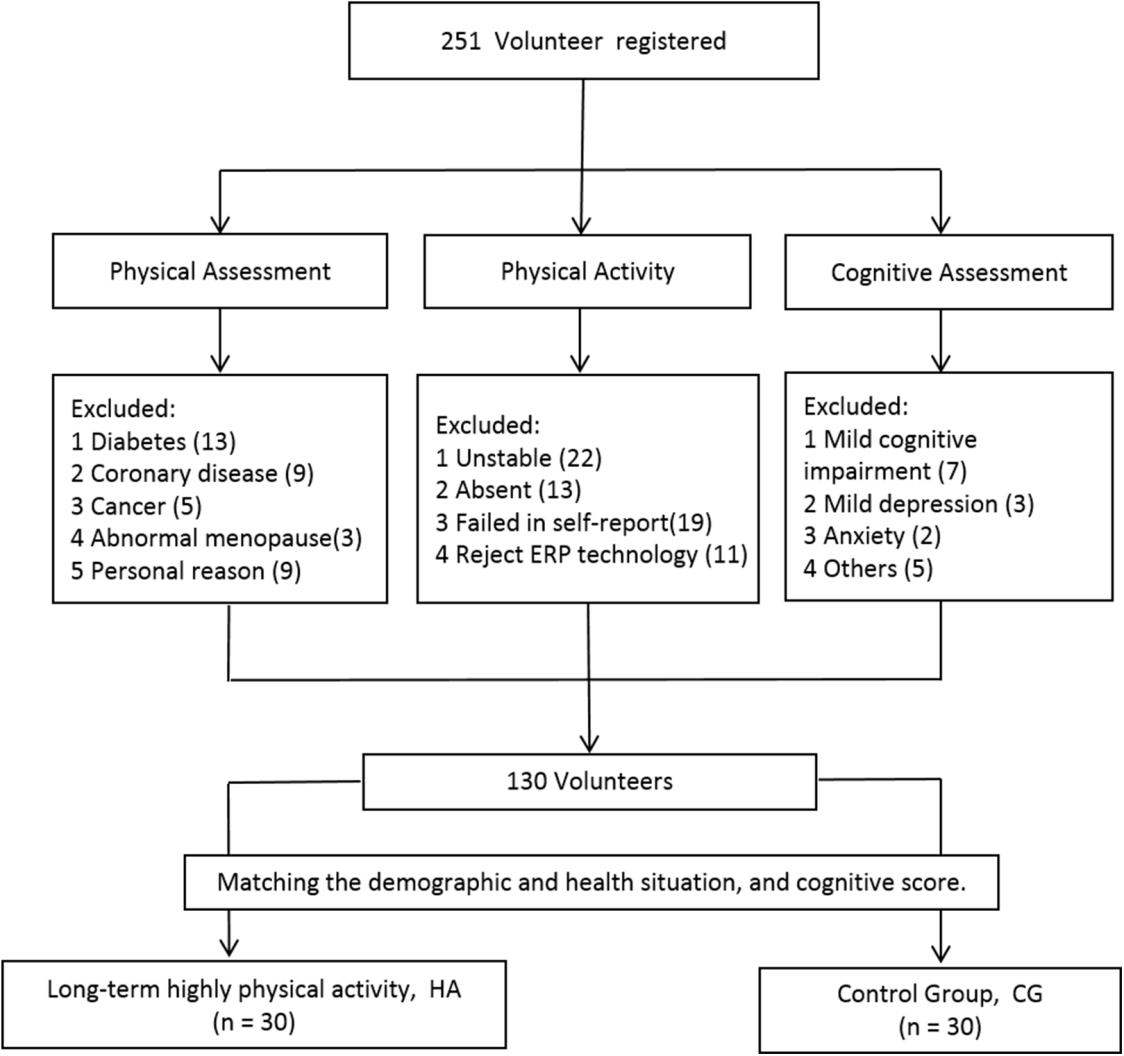
Screening and grouping the participants.

The enrolled participants were assessed by both psychological and physical surveys: the Montreal Cognitive Assessment and Raven’s Standard Progressive Matrices were used to assess cognitive abilities, and the International Physical Activity Questionnaire (IPAQ, Chinese version) was used to assess physical activity levels. The IPAQ was assessed twice with a 3-month interval to improve validity. Only those individuals with similar results on both rounds of the IPAQ and who had maintained the same level of activity for 3 or more years were enrolled in the formal study (Fig. 1).

In total, 60 participants were grouped according to their physical activity level (Bauman et al., 2009). Individuals in the long-term high physical activity group (*n* = 30) satisfied at least one of the following conditions: (1) more than 3 days of high intensity activity in a week, and gross metabolic equivalent (MET) minutes higher than 1,500 MET-minutes/week; or (2) gross METs higher than 3,000 MET-minutes/week obtained through walking or other moderate or high intensity activity. Participants who did reach the conditions required for joining the high activity group were placed in the control group (*n* = 30). Owing to potential influences of various factors, participants were selected on the basis of their demographic and health characteristics as well as their cognitive scores, and individuals in the two groups were matched on these aspects. Participant demographic characteristics are presented in Table 1.

**Table 1:** Baseline data of the participant characteristics (mean ± SD).

Variables	High active, HA	Control group, CG
*n*	30	30
Demographic and health data		
Age (year)	59.83 ± 3.39	59.10 ± 4.31
Menopause prior (year)	9.40 ± 4.27	9.20 ± 4.82
BMI (kg/cm^2^)	23.86 ± 3.44	24.68 ± 3.11
Cognitive score		
MoCA	26.10 ± 3.02	26.07 ± 2.74
SPM	68.83 ± 18.08	69.17 ± 20.47
Physical activity		
Gross MET	8294.97 ± 2462.64	2028.72 ± 540.17
High intensity (METs)	2022.67 ± 1873.04	–
VPA (min)	252.83 ± 234.13	–
Moderate intensity (METs)	4850.00 ± 2425.83	1164.67 ± 562.03
MVPA (min)	1212.50 ± 606.46	291.17 ± 140.51
Walking (METs)	1422.30 ± 990.82	864.05 ± 489.09

**Note:**

BMI, body mass index; MET, metabolic equivalent; VPA, value per action; MVPA, moderate to vigorous physical activity.

### Procedures

The experimental procedures used in the present study were similar to those used in previous ERP studies (Wang, Zhou & Chang, 2015; Yang et al., 2009). The participants were seated in an electrically shielded room, 100 cm away from a 13-inches Dell laptop. The cognitive measurement was designed and run by E-prime software. After the consent was given, participants were asked to perform the practice in order to familiar with the task. The practice contains 10 trials and followed with the formal task. Overall, introduction, familiarization, and the anticipation tests took approximately 30 min to complete.

### Cognitive measurement

**Figure 2:**
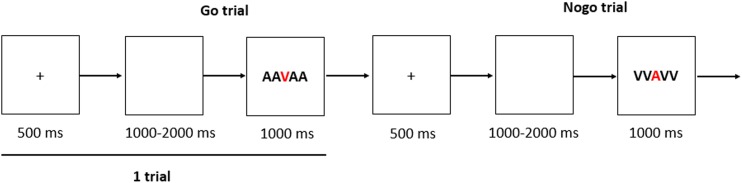
The sequence of events within a single trial of the task.

The cognitive measurement is a Go–No-go task, which consisted of 200 trials in all. Each trial begins with a 500 ms fixation followed by a white screen which duration varied randomly between 1,000 and 2,000 ms. Then the Go stimulus (“AAVAA”) or the No-go stimulus (“VVAVV”) would appear within 1,000 ms (Fig. 2). The participants were asked to press the “ENTER” key as quickly as possible when the Go stimulus appears and not press any key in the No-go trials, the response window will last 1,000 ms, unless the “ENTER” key is pressed by participants. Only one block in this task and the rate of Go trials verses No-go trials was 3:1, and no feedback was provided during the test trials.

### Electroencephalogram (EEG) recording and analysis

The EEG activity was collected using a 64-channel BrainAmp amplifier, with electrode positions based on the international 10–10 system, and recorded with the BrainVision Recorder system (Brain Products GmbH, Gilching, Germany). The EEG signals were recorded from Ag/AgCl electrodes attached to an electro-cap, with FCz and AFz serving as the online reference and ground electrodes, respectively. Two electrodes were placed on the mastoids for later re-referencing. A vertical eye-movement electrooculogram was recorded from electrodes placed just below the left eye. Horizontal eye-movement electrooculograms were recorded from electrodes placed at the outer canthus of the right eye. The EEG signals were amplified using a 0.01–100 Hz bandpass filter and digitized at a sampling rate of 500 Hz. All electrode impedances were maintained at less than 10 kΩ.

**Figure 3:**
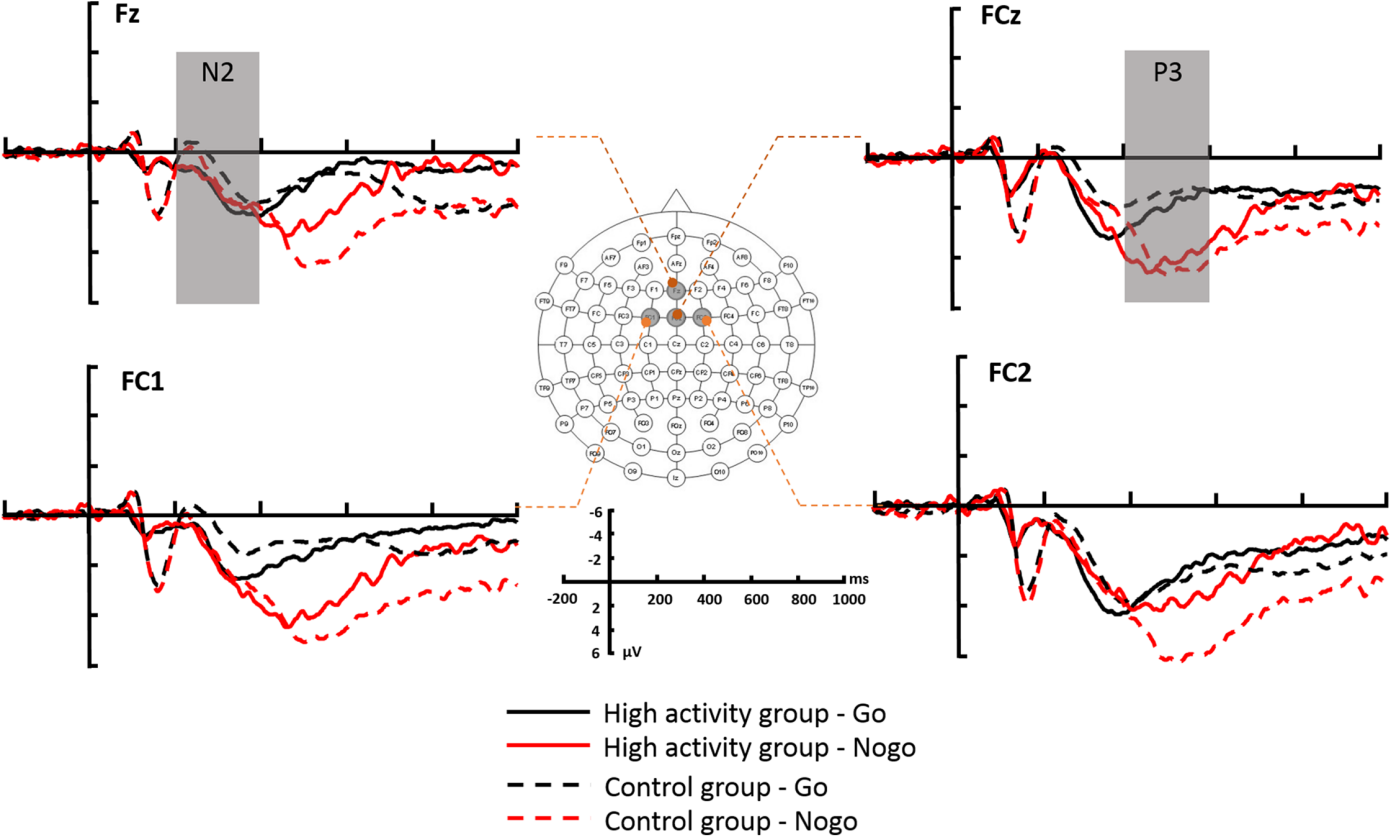
The grand-average ERPs at the Fz, FCz, FC1 and FC2 recording sites. HA, highly physical activity group; CG, control group.

All EEG data processing was conducted offline, using Brain Products Analyzer 2 software (Brain Products GmbH, Gilching, Germany). After the re-referencing was performed using the average of the TP9 and TP10 electrodes, the signals were bandpass filtered from 1 to 24 Hz. The ERPs were then acquired for the correct trials using stimulus-locked epochs from −200 to 1,000 ms according to the type of trial (Go vs No-go). The 200-ms prestimulus period was used for baseline correction. Trials with amplitudes exceeding ±100 μV were excluded for these contaminating artifacts. The N2 peak amplitude and latency were recorded from the Fz, FCz, FC1, and FC2 electrodes within a 200–400 ms time window, and the peak amplitude and latency of the P3 component were recoded from the same electrodes within 400–600 ms of stimulus onset. The electrode selection was based on the grand-average waveforms (Fig. 3) in the present study combined with previous findings (Benvenuti et al., 2015; Vallesi et al., 2009; Zhang, Xu & Chang, 2016).

### Data analysis

Statistical analyses were performed using SPSS Statistics 20.0 (IBM, Somers, NY, USA). Descriptive data are presented as the mean ± SD. The significance level was set at 0.05. Independent *t*-tests were performed on measurements of behavioral data, with group (high activity or control) as independent factors and the Go reaction time (Go RT) and accuracy, and the No-go accuracy as dependent factors. The Go RT is calculated as the mean reaction time with the correct responses in all the Go trials, to represent the speed performance, and the accuracies are calculated with the correct response or inhibition in the Go or No-go trials respectively, which represent as the accuracy performance.

For the neuroelectrophysiologic data, three-way repeated measures analysis of variance (ANOVA) was performed separately for the N2 amplitude, N2 latency, P3 amplitude, and P3 latency, and with trial type (Go or No-go) and electrode (Fz, FCz, FC1, or FC2) as within-subjects factors and group (high activity or control) as between-subjects factors.

The Greenhouse–Geisser epsilon correction was applied to adjust the degrees of freedom of the *F* ratios if sphericity was violated. Post hoc testing of significant main effects was conducted using the Bonferroni method. Significant interactions were analyzed using a simple effects model. Partial eta-squared (η_*p*_^2^) was reported to demonstrate the effect size in the ANOVA tests.

## Results

### Behavioral results

For the Go RT, a significant difference was found between groups, t(58) = −5.118, *p* < 0.001. The high activity group (475.04 ± 34.09 ms) had a faster Go RT than the control group (542.11 ± 63.17 ms) (Table 2).

**Table 2:** Behavioral data for the standard Go/Nogo task (mean ± SD).

Variables	High active	Control group
Go RT (ms)	475.04 ± 34.09	542.11 ± 63.17
Go accuracy (%)	99.23 ± 1.70	98.20 ± 2.59
Nogo accuracy (%)	96.87 ± 4.26	94.33 ± 6.30

There was no significant difference between the two groups on the Go trial accuracy (t_(58)_ = 1.824, *p* = 0.074), and No-go trial accuracy (t_(58)_ = 1.626, *p* = 0.109).

### ERP results

#### N2 component

**Figure 4:**
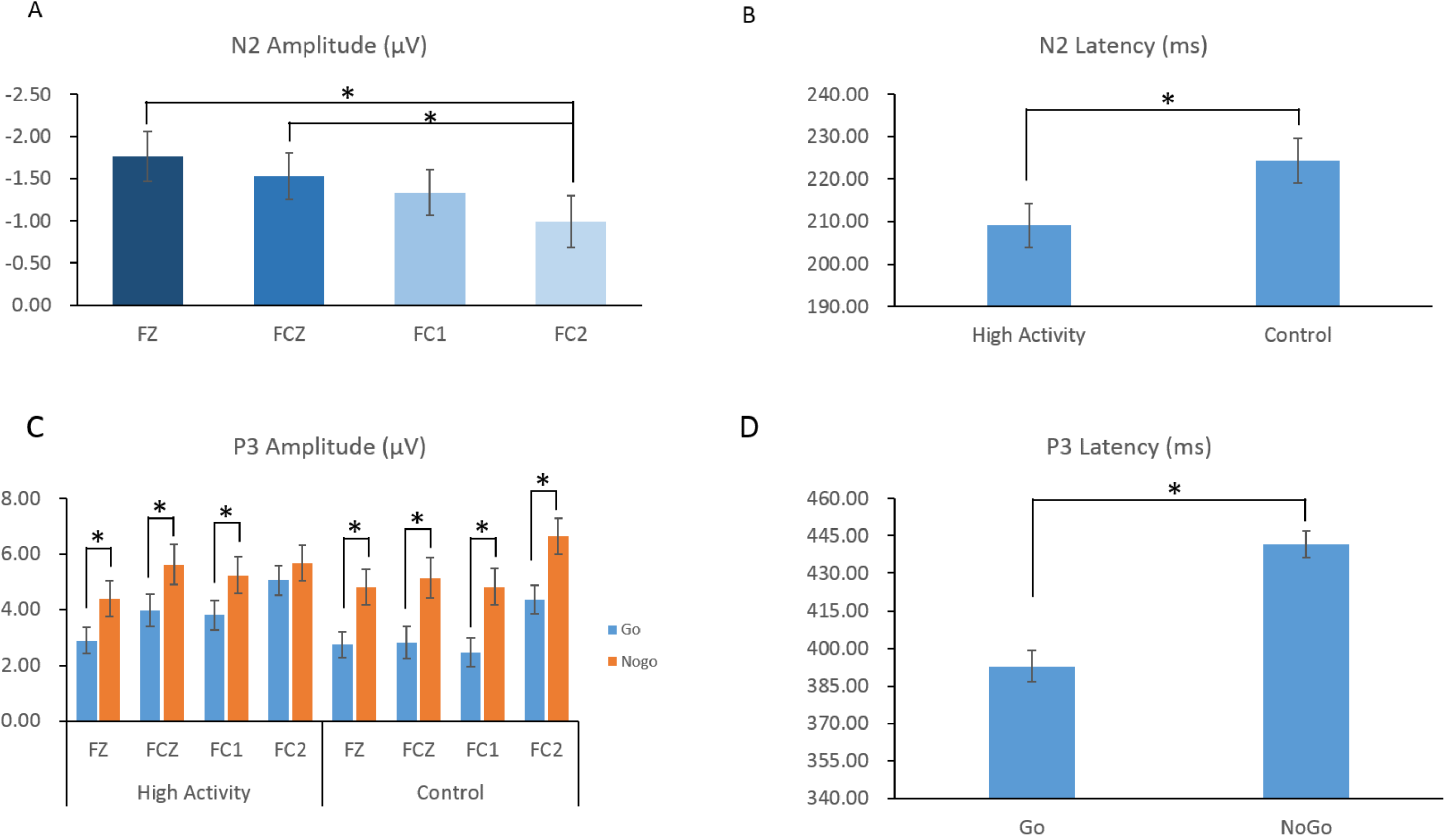
The amplitudes (A) and latency (B) of N2 and P3 components in response to the go and no-go trials across groups. The asterisk symbol (*) indicates that the statistical significance was *p* < 0.05. HA, highly physical activity group; CG, control group.

For the N2 amplitude, the main effect of electrode was significant (*F*_(3,174)_ = 6.553; *p* < 0.001; η_*p*_^2^ = 0.102) (Fig. 4A). The N2 amplitude was largest at electrode FC2 (−0.99 ± 2.34 μV), which was significantly larger than at Fz (−1.76 ± 2.26 μV) (*p* = 0.001) or FCz (−1.53 ± 2.12 μV) (*p* = 0.011). There was no significant results in the main effect of trial type (*F*_(1,58)_ = 0.041; *p* = 0.839; η_*p*_^2^ = 0.001), group (*F*_(1,58)_ = 0.243; *p* = 0.624; η_*p*_^2^ = 0.004), and the interaction between trial type, electrode, and group (*F*_(3,174)_ = 0.132; *p* = 0.941; η_*p*_^2^ = 0.002) (Table 3).

**Table 3:** ERP data (amplitude and latency) for the standard Go/Nogo task (mean ± SD).

Variables	High active		Control group	
ERP results	Amplitude (μV)	Latency (ms)	Amplitude (μV)	Latency (ms)
Go-N2				
FZ	−1.84 ± 1.82	207.27 ± 44.46	−1.70 ± 3.05	218.30 ± 40.59
FCZ	−1.54 ± 2.17	218.63 ± 34.10	−1.57 ± 2.69	227.80 ± 45.62
FC1	−1.48 ± 2.17	202.83 ± 38.29	−1.18 ± 2.55	239.10 ± 61.65
FC2	−0.90 ± 2.61	206.07 ± 38.89	−1.15 ± 3.07	220.43 ± 39.95
Nogo-N2				
FZ	−1.99 ± 2.61	208.37 ± 41.83	−1.51 ± 1.96	212.83 ± 41.81
FCZ	−1.71 ± 2.20	222.40 ± 41.60	−1.30 ± 1.79	229.20 ± 40.74
FC1	−1.73 ± 2.23	207.13 ± 37.92	−0.94 ± 1.74	226.13 ± 31.98
FC2	−1.08 ± 2.39	199.93 ± 38.64	−0.84 ± 1.96	220.40 ± 35.98
Go-P3				
FZ	2.89 ± 2.19	398.23 ± 64.03	2.74 ± 2.81	401.63 ± 61.28
FCZ	3.97 ± 3.24	387.97 ± 54.57	2.82 ± 2.92	384.67 ± 61.42
FC1	3.80 ± 3.26	390.63 ± 70.36	2.47 ± 2.35	396.10 ± 64.78
FC2	5.05 ± 2.99	383.70 ± 49.60	4.36 ± 2.73	399.93 ± 54.33
Nogo-P3				
FZ	4.39 ± 3.61	424.87 ± 61.26	4.81 ± 3.45	453.57 ± 57.75
FCZ	5.62 ± 3.89	450.20 ± 45.33	5.14 ± 4.02	450.30 ± 59.39
FC1	5.23 ± 3.73	426.63 ± 60.69	4.82 ± 3.45	450.70 ± 53.33
FC2	5.67 ± 3.70	422.93 ± 53.62	6.63 ± 3.21	453.36 ± 56.42

For the N2 latency, the main effect of group was significant (*F*_(1,58)_ = 4.309; *p* = 0.042; η_*p*_^2^ = 0.069). The N2 latency in the high activity group (209.08 ± 27.14 ms) was shorter than that in control group (224.28 ± 29.52 ms). The main effect of electrode was also significantly different (*F*_(3,174)_ = 3.306; *p* = 0.027; η_*p*_^2^ = 0.054) (Fig. 4B). The N2 latency at the FC2 electrode (211.71 ± 36.52 ms) was shorter than that at the Fz electrode (211.69 ± 38.99 ms), but not reach significant (*p* = 0.060). However, the main effect of trial type (*F*_(1,58)_ = 0.399; *p* = 0.530; η_*p*_^2^ = 0.007), and the interaction between trial type, electrode, and group (*F*_(3,174)_ = 1.907; *p* = 0.130; η_*p*_^2^ = 0.032) were not significant. In order to comparing with the behavior results, we did the t-tests between group for Go and No-go trial types separately. The only significant result was found in the Go trial type, that the high activity group (208.700 ± 29.51 ms) had a shorter N2 latency than the control group (226.408 ± 34.51 ms).

#### P3 component

For the P3 amplitude, the main effect of trial type was significant (*F*_(1,58)_ = 46.836; *p* < 0.001; η_*p*_^2^ = 0.447). The P3 amplitude during Go trials (3.51 ± 2.63 μV) was smaller than that during No-go trials (5.39 ± 3.34 μV). There was also a main effect of electrode (*F*_(3,174)_ = 17.662; *p* < 0.001; η_*p*_^2^ = 0.233). The P3 amplitude was largest at FC2 (5.43 ± 2.95 μV) and smallest at Fz (3.71 ± 2.78 μV). However, the main effect of group was not significant (*F*_(1,58)_ = 0.234; *p* = 0.630; η_*p*_^2^ = 0.004).

These main effects, however, were superseded by significant interactions between trial type and electrode (*F*_(3,174)_ = 3.886; *p* = 0.010; η_*p*_^2^ = 0.063), trial type and group (*F*_(1,58)_ = 4.570; *p* = 0.038; η_*p*_^2^ = 0.072), and trial type, electrode, and group (*F*_(3,174)_ = 2.898; *p* = 0.037; η_*p*_^2^ = 0.048). Further analysis showed that for the control group, the P3 amplitude on No-go trials was larger than that on Go trials at all electrodes (*p values* < 0.001). Meanwhile, for the high activity group, the P3 amplitude on No-go trials was larger than that on Go trials at all electrodes (*p values* < 0.050) with the exception of the FC2 site in which there was no difference (Fig. 4C).

For the P3 latency, the main effect of trial type was significant (*F*_(1,58)_ = 69.601; *p* < 0.001; η_*p*_^2^ = 0.545). The P3 latency on Go trials (392.86 ± 47.85 ms) was significantly shorter than that on No-go trials (441.57 ± 43.20 ms) (Fig. 4D). However, the main effect of group (*F*_(1,58)_ = 1.679; *p* = 0.200; η_*p*_^2^ = 0.028), electrode (*F*_(3,174)_ = 0.264; *p* = 0.851; η_*p*_^2^ = 0.005), and the interaction between trial type, electrode, and group (*F*_(3,174)_ = 0.395; *p* = 0.757; η_*p*_^2^ = 0.007) were not significant.

## Discussion

In the present study, behavioral performance and ERP components were assessed during performance on a visual Go/No-go task among postmenopausal women with long-term high physical activity and less physically active age-, cognition-, and educational level-matched controls. The effects of long-term high physical activity on behavioral and electrophysiologic measures during task performance were analyzed. At the behavioral level, a shorter Go RT was found for women in the long-term high physical activity group compared with those in the control group. At the electrophysiologic level, a shorter N2 latency was observed in the long-term high physical activity group in the Go trials, which was consistent with the behavioral result.

Besides, the current study also found a larger P3 amplitude in the frontocentral brain region for both groups, during the performance of No-go trials than Go trials, except in the FC2 sites for high physical activity group. These results mostly are similar to prior studies that reported the benefits of physical activity to the inhibitory control (Boucard et al., 2012). However, the current study only focused on the postmenopausal women with long-term high physical activity and less physical activity.

The behavioral data indicated that the long-term high activity group reacted faster than the control group during the Go trials, whereas no significant group difference was found in the No-go trials. This result is consistent with previous studies that reported fitness-related performance differences in reaction time tasks, showing shorter reaction times in higher-fit individuals compared with lower-fit individuals (Spirduso, 1975). Also, in a previous intervention study, after a 10-week regular exercise program, the aged individuals showed improved performance in reaction time, while a group of nonexercisers showed no improvements (Lord & Castell, 1994). With more physical activity, postmenopausal women may improve reaction time not only in a simple reaction time task (Hultsch, MacDonald & Dixon, 2002) but also in an inhibition control–relevant task. This lack of a significant effect of physical activity on error rate may have been caused by a ceiling effect in the behavioral performance (Stroth et al., 2009).

The N2 component results revealed patterns similar to those associated with behavioral inhibition. A shorter N2 latency was found in the long-term high activity group in the current study. Consistent with the behavioral results, the shorter N2 latency indicated that physical activity increased the efficiency of inhibitory control system by improving the activity of response monitoring processes. Previous studies have consistently shown that N2 recorded from frontocentral electrodes is a major and sensitive ERP marker of inhibitory processes (Falkenstein, 2006; Folstein & Van Petten, 2008). Thus, our finding supports the beneficial effect of physical activity, with shortened N2 latencies at frontocentral sites for the high physical activity group. Furthermore, the further analysis on the main effect of Electrode showed that the FC2 position had a larger N2 amplitude and a shorter latency than other frontal and central regions. However, this hemisphere result requires confirmation in future studies.

For the P3 component, a significant difference between the long-term high activity and control groups was detected as an interaction between electrode, trial type, and group. In the control group, performance on a No-go trial elicited greater P3 peak amplitudes than did performance on a Go trial in all the frontal and frontocentral brain regions. Similarly, the long-term high activity group also showed the difference between trial types, but no such difference in the FC2 position. Previous studies suggested that in the Go/No-go task, the P3 reflects both cognitive and motor inhibition, and regarded as the movement-related potentials (Verleger et al., 2006; Bokura, Yamaguchi & Kobayashi, 2001). Meanwhile, some functional MRI studies suggested the right hemisphere dominated the inhibitory control (Garavan, Ross & Stein, 1999; Kaufman et al., 2003), such the right hemisphere could be a sensitive to the Go/No-go task for the postmenopausal women. However, the current study only used ERP, and more further studies are needed to investigate this result. In addition, a larger P3 peak amplitude and longer P3 latency were found in No-go than in Go trial performances. The P3 component is assumed to reflect attentional stimulus evaluation processes (Donchin & Coles, 1988), and the latency of P3 has been related to stimulus classification speed (Polich, 2007). Previous studies have shown that there is a greater P3 amplitude or a shorter P3 latency in highly fit individuals compared with lower-fit individuals, suggesting that physical activity leads to enhanced attentional resource allocation or shorter information processing time (Chu et al., 2015; Drollette et al., 2014).

The present study investigated the beneficial effects of physical exercise on inhibitory control among a sample population of postmenopausal women, and there are a few potential limitations that should be addressed. The self-reported physical activity data used in the present study may increase the subjectivity of assessment compared with data obtained through the use of an accelerometer (Dyrstad et al., 2014). In addition, the lack of a significant difference observed in the accuracy performance between the two groups might be the ceiling effect in the behavioral performance. Future studies should examine tasks with a higher workload or greater difficulty to determine whether relevant potential effects in cognitive performance exist. Finally, the aerobic level, and the nutrition supplements, including the Vitamin and Calcium, and potential drug in their daily medical pills, which might influence the inhibitory control (Edwards et al., 2011), should be considered as the covariations in the further studies.

## Conclusions

The present study provides evidence in support of the beneficial role of long-term high physical activity on cognitive inhibition in postmenopausal women. The more physically active women exhibited faster reaction times than the less active women during the performance of a task that taps into inhibition processing. However, we did not find out any significant difference between groups to the inhibitory control (i.e., No-go trials), which might be some potential factors, such as the task difficulty. Such, our preliminary findings gave some information to the relationship between physical activity and inhibitory control in postmenopausal women and suggest that further examination of these effects in specific aged populations is warranted.

## Supplemental Information

10.7717/peerj.6523/supp-1 Supplemental Information 1 The amplitudes of N2 and P3 components in group 1 and group 2.

10.7717/peerj.6523/supp-2 Supplemental Information 2 Raven’s standard progressive matrices (RSPM).

10.7717/peerj.6523/supp-3 Supplemental Information 3 Montreal cognitive assessment (MOCA).

10.7717/peerj.6523/supp-4 Supplemental Information 4 International physical activity questionnaire (IPAQ).

## Additional Information and Declarations

### Data Availability

The following information was supplied regarding data availability: Raw data is available in a Supplemental File.
