# Supplementary material for: Long-term high physical activity modulates event-related potential indices of inhibitory control in postmenopausal women
Source: PeerJ. 2019 Mar 18;7:e6523. doi: 10.7717/peerj.6523 (PMC6428037; doi:10.7717/peerj.6523)
Supplement: Supplemental Information 2 [file peerj-07-6523-s002.docx]

**Raven's Standard Progressive Matrices**

**ID:______ Name:__________ Date:___________**

**Illustration:** The following pictures have their own themes, but each of them lacks a part. Each theme picture has six selections , please find the perfect one to match the theme one and make the whole picture rational and complete.

| 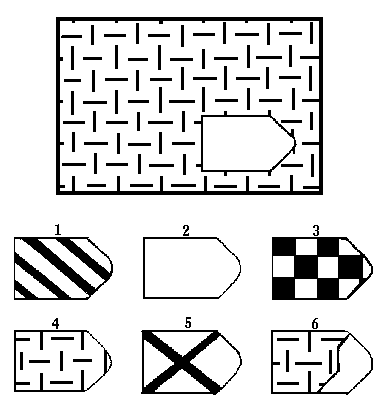  A1 | 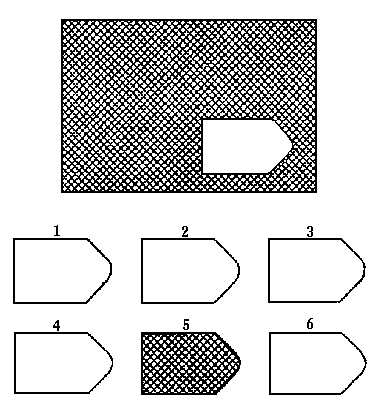  A2 |
| --- | --- |
| 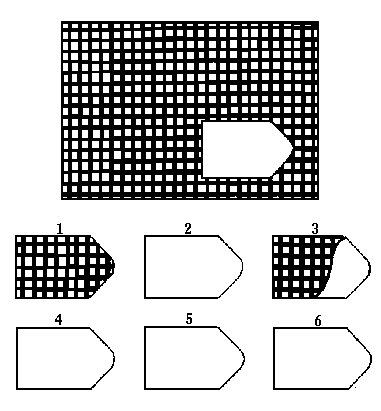  A3 | 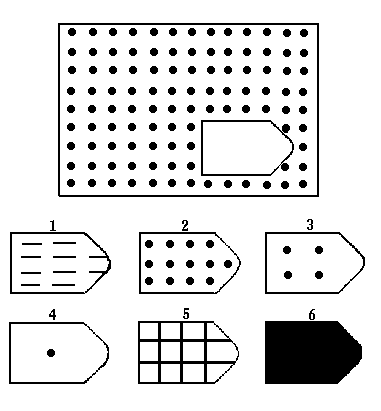  A4 |
| 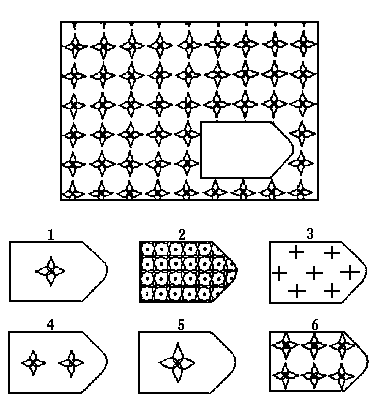  A5 | 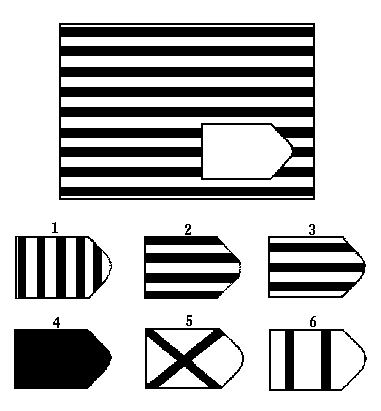  A6 |
| 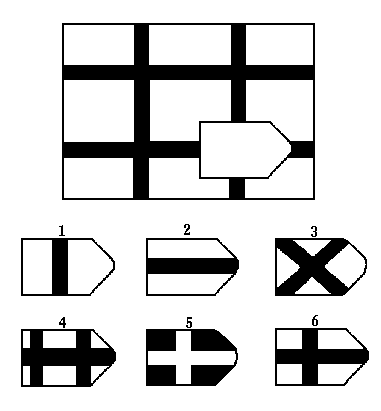  A7 | 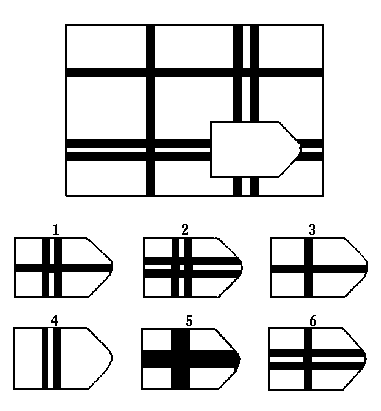  A8 |
| 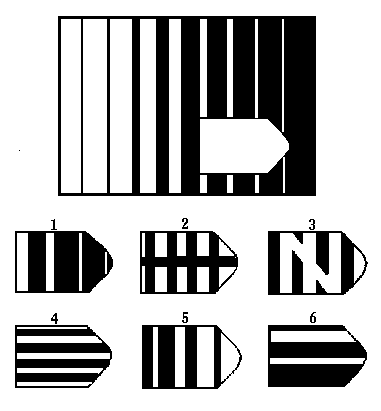  A9 | 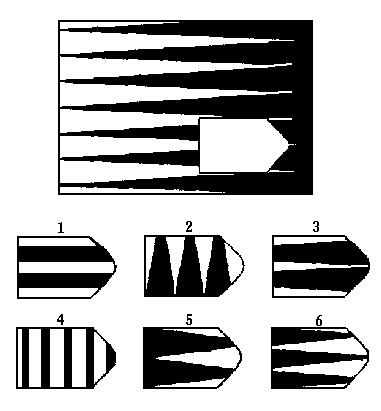  A10 |
| 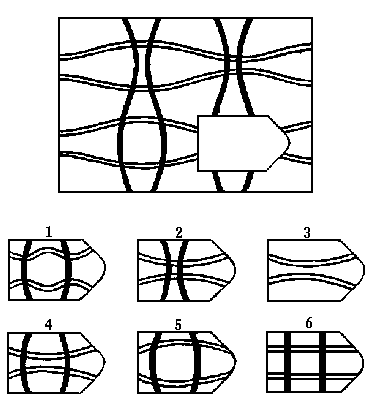  A11 | 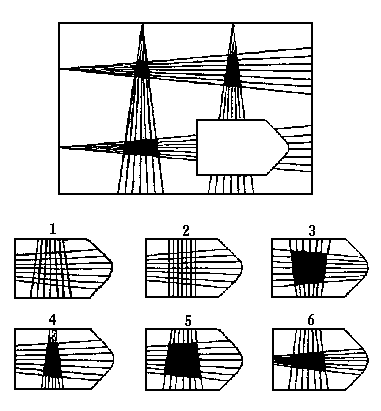  A12 |

| 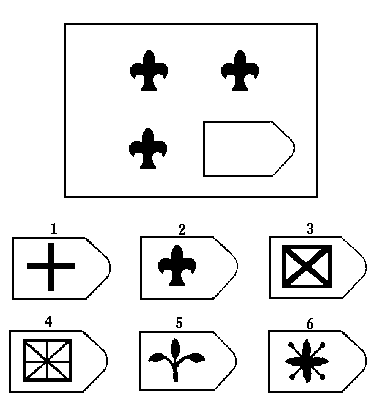  B1 | 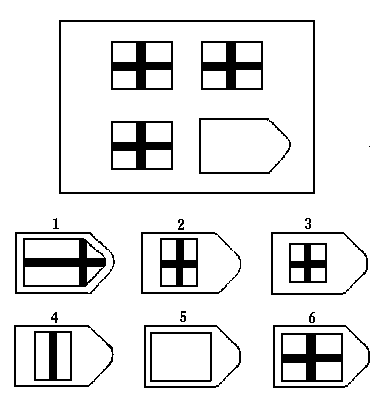  B2 |
| --- | --- |
| 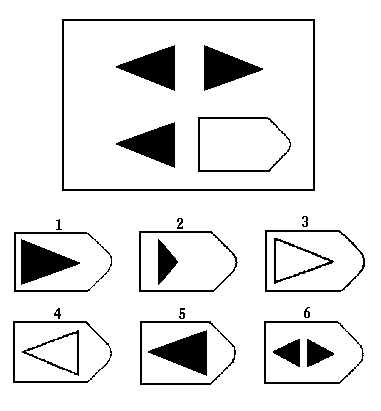  B3 | 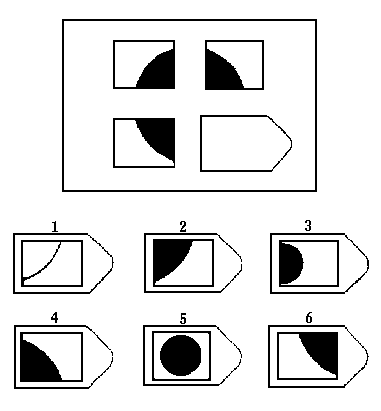  B4 |
| 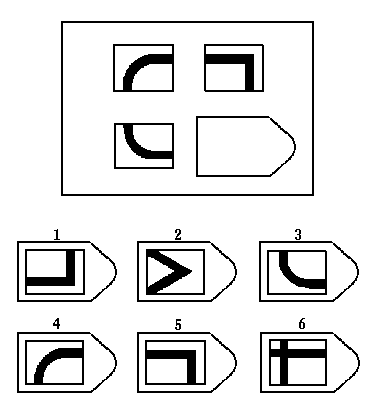  B5 | 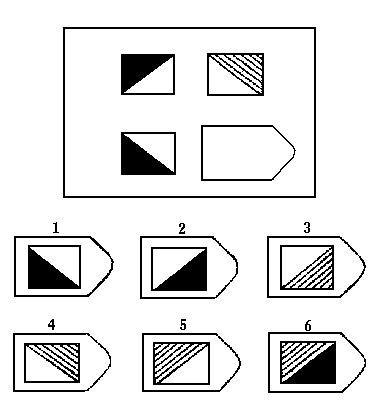  B6 |

| 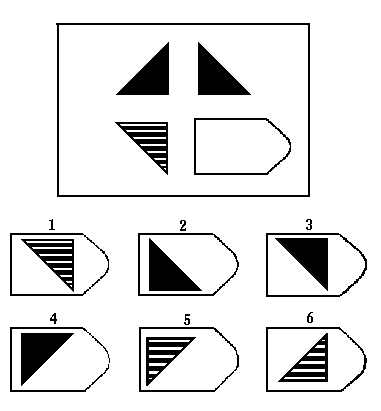  B7 | 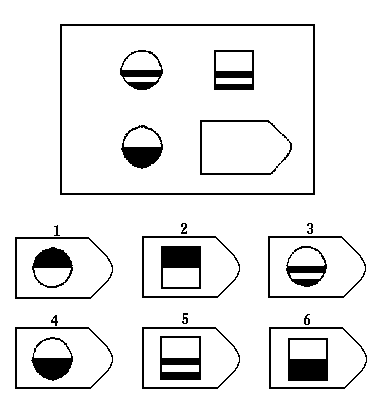  B8 |
| --- | --- |
| 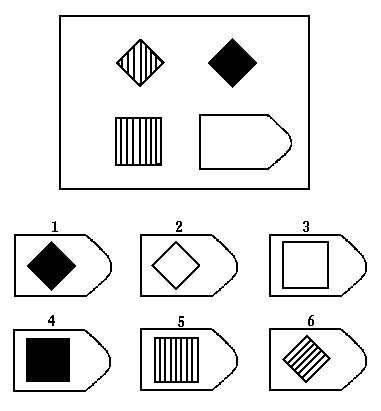  B9 | 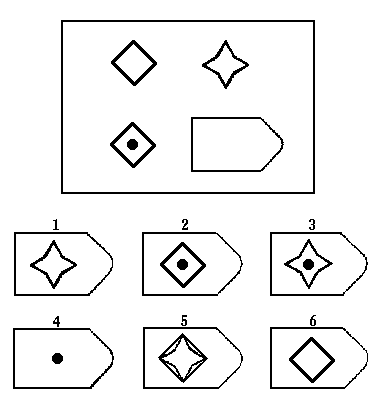  B10 |
| 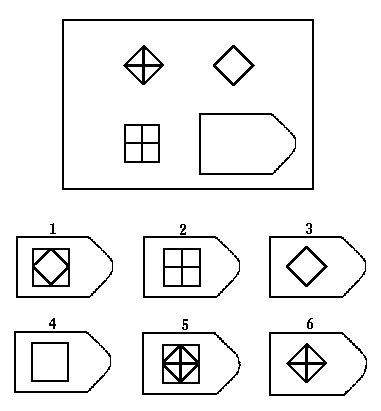  B11 | 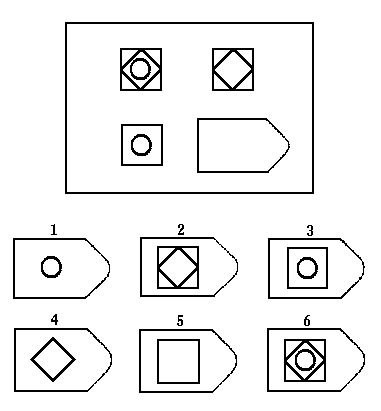  B12 |

| 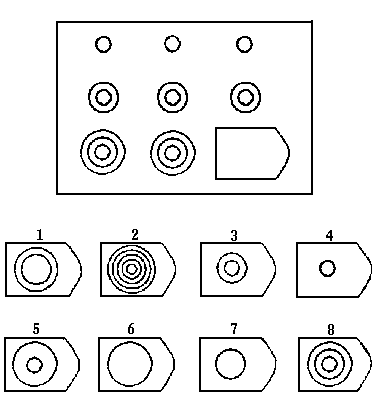  C1 | 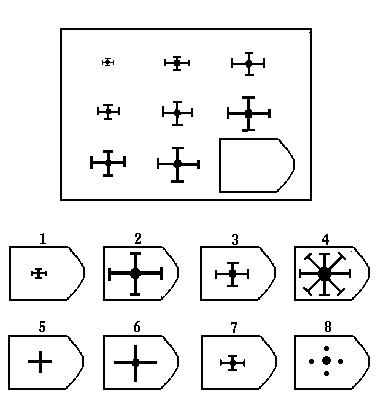  C2 |
| --- | --- |
| 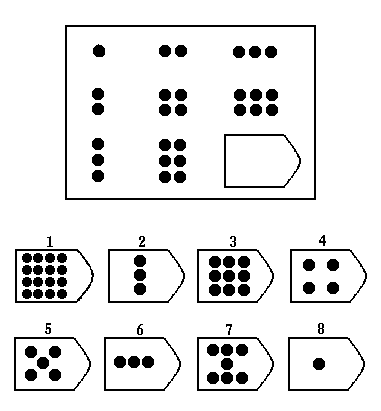  C3 | 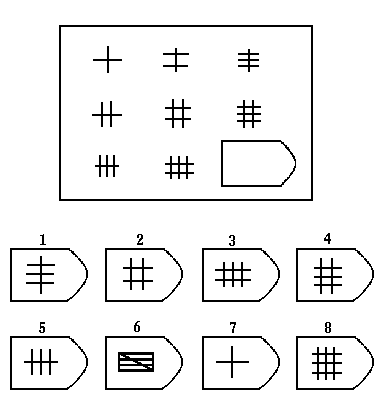  C4 |
| 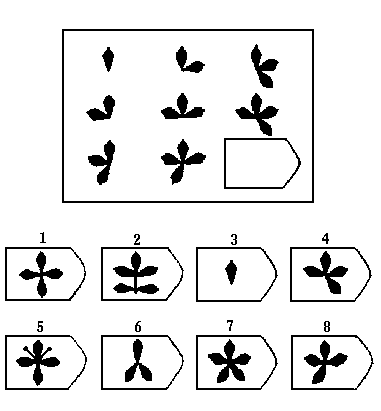  C5 | 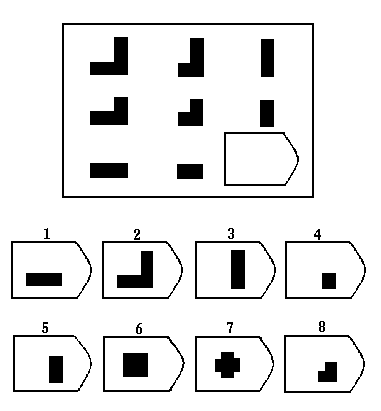  C6 |

| 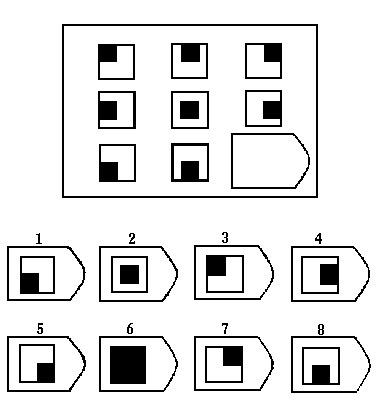  C7 | 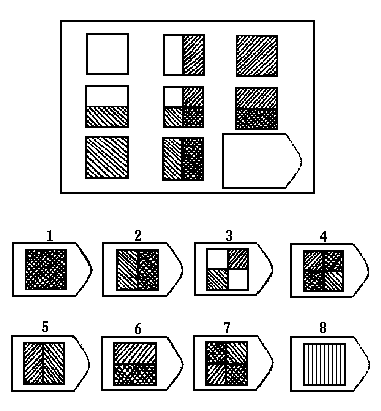  C8 |
| --- | --- |
| 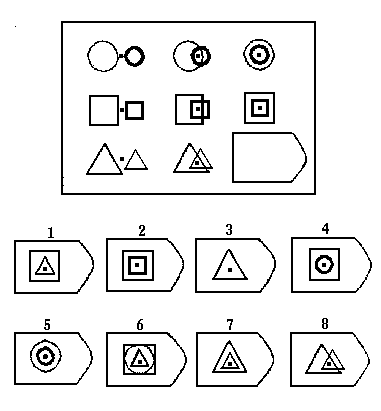  C9 | 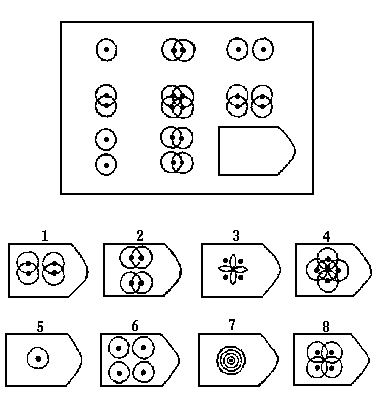  C10 |
| 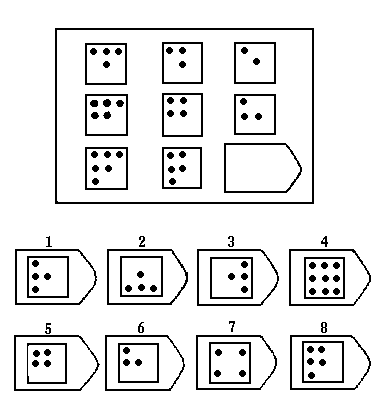  C11 | 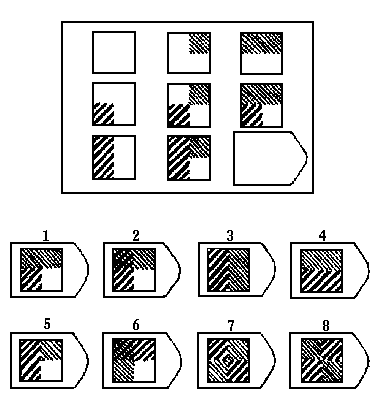  C12 |

| 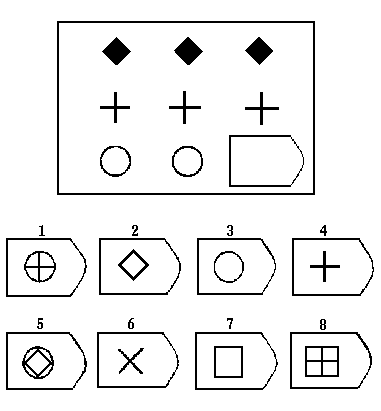  D1 | 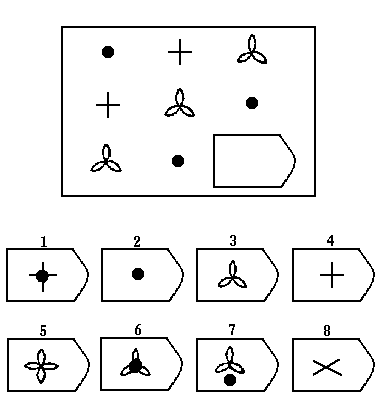  D2 |
| --- | --- |
| 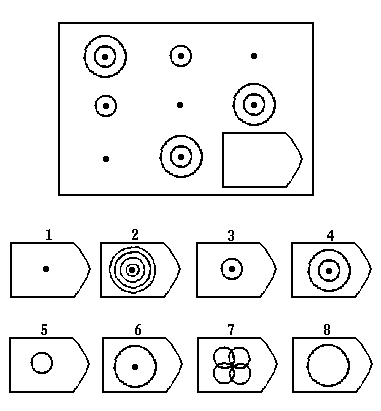  D3 | 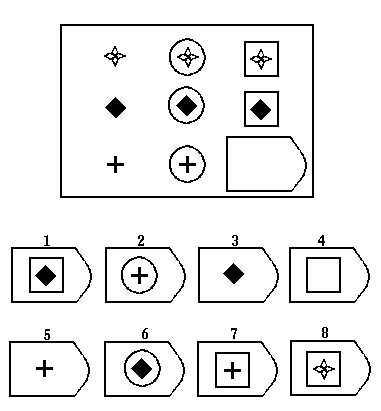  D4 |
| 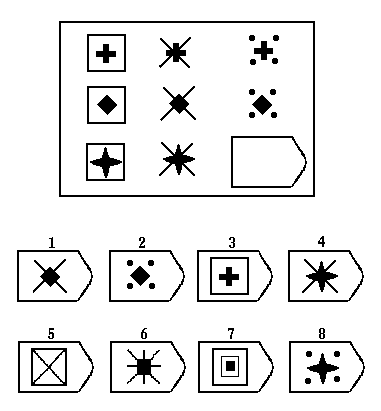  D5 | 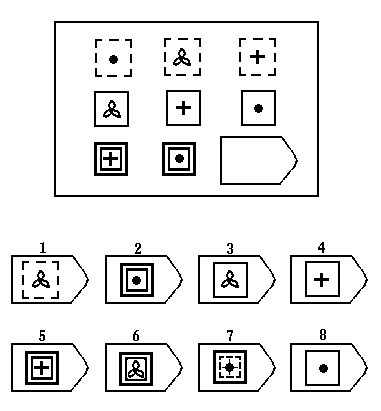  D6 |

| 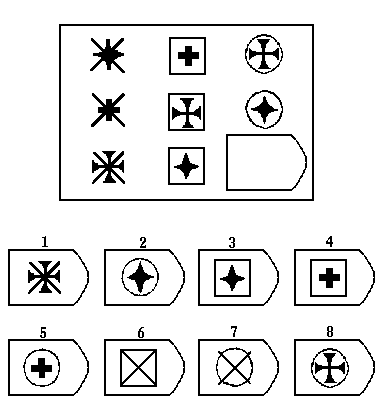  D7 | 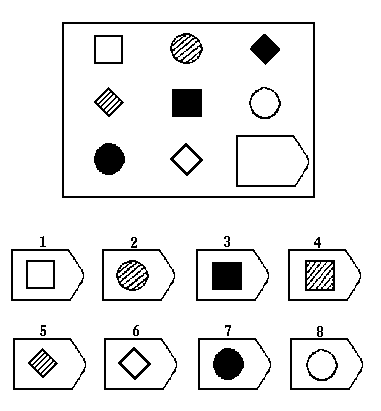  D8 |
| --- | --- |
| 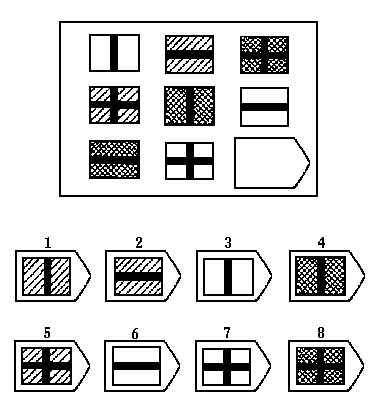  D9 | 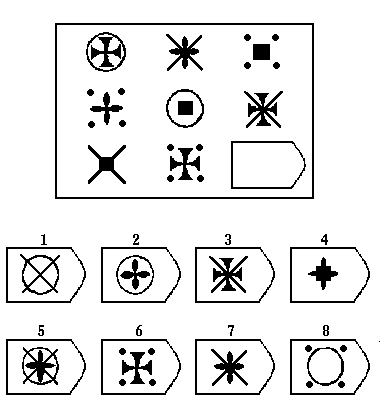  D10 |
| 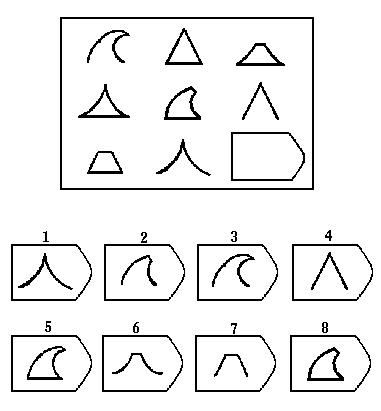  D11 | 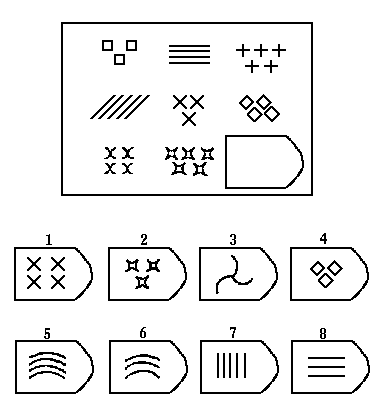  D12 |

| ***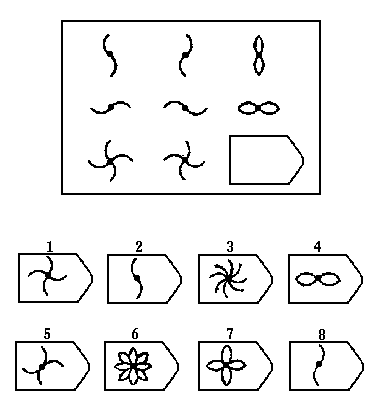***  E1 | ***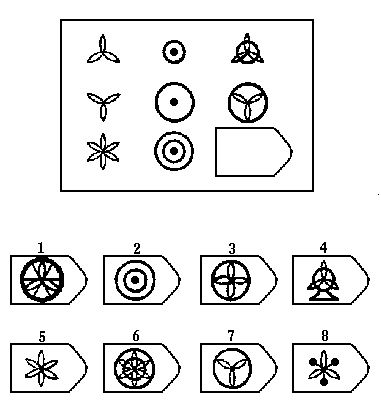***  E2 |
| --- | --- |
| ***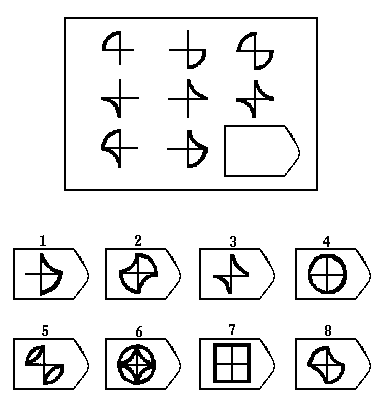***  E3 | ***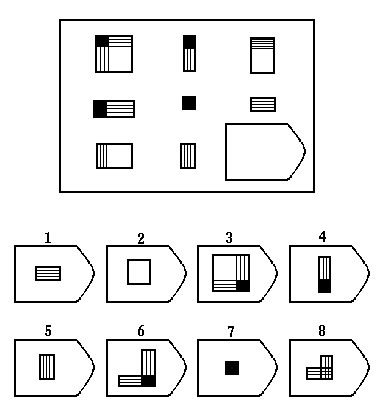***  E4 |
| ***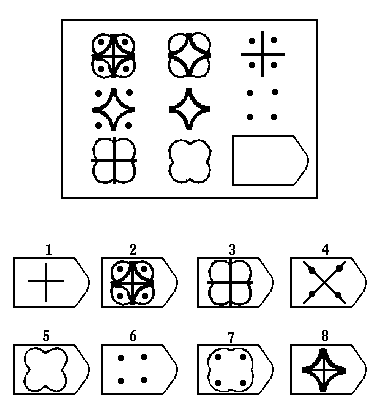***  E5 | ***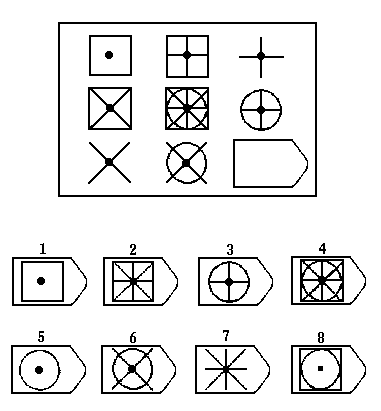***  E6 |

| ***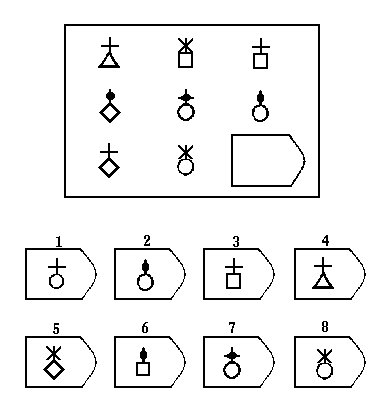***  E7 | ***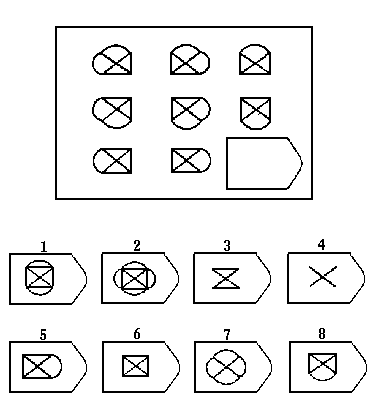***  E8 |
| --- | --- |
| ***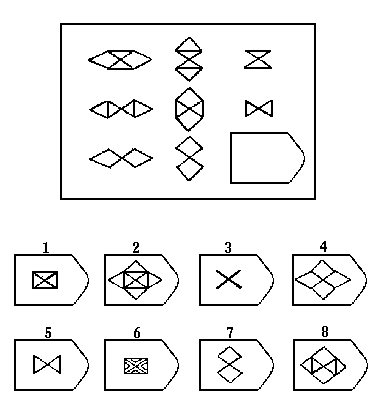***  E9 | ***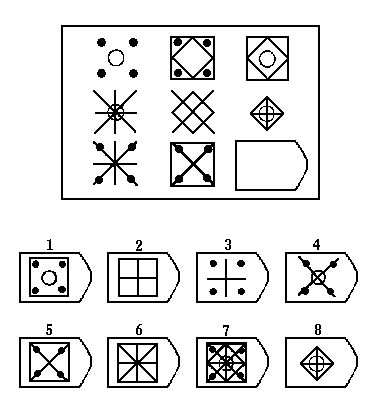***  E10 |
| ***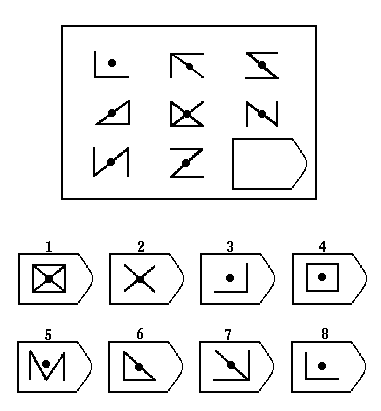***  E11 | ***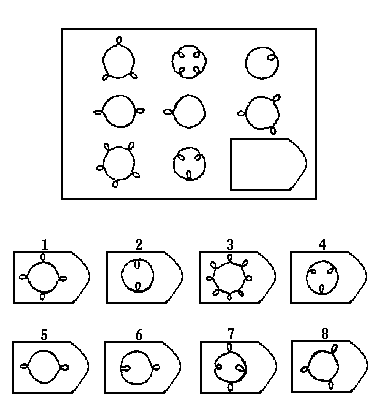***  E12 |

Raven's Standard Progressive Matrices

**(Calculating points,Conversion standard, Assessment)**

**1、Calculating points: One point for each question, and the fraction of each part is added to the total score.**

Raven's Standard Progressive Matrices Reference Answers

┏━┯━┯━┯━┯━┯━┯━┯━┯━┯━┯━┯━┯━┓
┃　│1 │2 │3 │4 │5 │6 │7 │8 │9 │10│11│12┃
┠─┼─┼─┼─┼─┼─┼─┼─┼─┼─┼─┼─┼─┨
┃Ａ│4 │5 │1 │2 │6 │3 │6 │2 │1 │3 │4 │5 ┃
┠─┼─┼─┼─┼─┼─┼─┼─┼─┼─┼─┼─┼─┨
┃Ｂ│2 │6 │1 │2 │1 │3 │5 │6 │4 │3 │4 │5 ┃
┠─┼─┼─┼─┼─┼─┼─┼─┼─┼─┼─┼─┼─┨
┃Ｃ│8 │2 │3 │8 │7 │4 │5 │1 │7 │6 │1 │2 ┃
┠─┼─┼─┼─┼─┼─┼─┼─┼─┼─┼─┼─┼─┨
┃Ｄ│3 │4 │3 │7 │8 │6 │5 │4 │1 │2 │5 │6 ┃
┠─┼─┼─┼─┼─┼─┼─┼─┼─┼─┼─┼─┼─┨
┃Ｅ│7 │6 │8 │2 │1 │5 │1 │6 │3 │2 │4 │5 ┃
┗━┷━┷━┷━┷━┷━┷━┷━┷━┷━┷━┷━┷━┛

**2.Conversion standard**

**Raven's Standard Progressive Matrices Scores and Percentile Rank Conversion Table**

| **Age**  **Year(s)** | **Standard Scores(%)**  **95 　90 　75 　50 　25 　10 　5** | **Age**  **Year(s)** |
| --- | --- | --- |
| **5.5**  **6**  **6.5**  **7**  **7.5**  **8**  **8.5**  **9**  **9.5**  **10**  **10.5**  **11**  **11.5**  **12**  **12.5**  **13**  **13.5**  **14**  **14.5**  **15**  **15.5**  **16**  **16.5**  **17**  **20**  **30**  **40**  **50**  **60**  **70** | **34 　29 　25 　16 　13 　12 　9**  **36 　31 　25 　17 　13 　12 　9**  **37 　31 　25 　18 　13 　12 　10**  **43 　36 　25 　19 　13 　12 　10**  **44 　38 　31 　21 　13 　12 　10**  **44 　39 　31 　23 　15 　13 　10**  **45 　40 　33 　29 　20 　14 　12**  **47 　43 　37 　33 　25 　14 　12**  **50 　47 　39 　35 　27 　17 　13**  **50 　48 　42 　35 　27 　17 　13**  **50 　49 　42 　39 　32 　25 　18**  **52 　50 　43 　39 　33 　25 　19**  **53 　50 　45 　42 　35 　25 　19**  **53 　50 　46 　42 　37 　27 　21**  **53 　52 　50 　45 　40 　33 　28**  **53 　52 　50 　45 　40　 35 　30**  **54 　52 　50 　46 　42 　35 　32**  **55 　52 　50 　48 　43 　36 　34**  **55 　53 　51 　48　 43　 36　 34**  **57 　54 　51 　48 　43 　36 　34**  **57 　55 　52 　49 　43 　41 　34**  **57 　56 　53 　49 　44 　41 　36**  **57 　56 　53 　49 　45 　41 　37**  **58 　57 　55 　52 　47 　40 　37**  **57 　56 　54 　50 　44 　38 　33**  **57 　55 　52 　48 　34 　37 　28**  **57 　54 　50 　47 　41 　31 　28**  **54 　52 　48 　42 　34 　24 　21**  **54 　52 　46 　37 　30 　22 　19**  **52 　49 　44 　33 　26 　18 　17** | **5.5**  **6**  **6.5**  **7**  **7.5**  **8**  **8.5**  **9**  **9.5**  **10**  **10.5**  **11**  **11.5**  **12**  **12.5**  **13**  **13.5**  **14**  **14.5**  **15**  **15.5**  **16**  **16.5**  **17**  **20**  **30**  **40**  **50**  **60**  **70** |
| **Age**  **Year(s)** | **95 　90 　75 　50 　25 　10 　5** | **Age**  **Year(s)** |

**2. Assess Intelligence**

**Intelligence Rank Reference**

| **Standard scores(%)** | **Rank** | **Intelligence** |
| --- | --- | --- |
| **≥９５** | **1** | **Excellent** |
| **７５～９４** | **2** | **Good** |
| **２５～７４** | **3** | **Average** |
| **５～２４** | **4** | **Below the average** |
| **〈５** | **5** | **Low level** |
