# Supplementary material for: Long-term high physical activity modulates event-related potential indices of inhibitory control in postmenopausal women
Source: PeerJ. 2019 Mar 18;7:e6523. doi: 10.7717/peerj.6523 (PMC6428037; doi:10.7717/peerj.6523)
Supplement: Supplemental Information 3 [file peerj-07-6523-s003.docx]

**Montreal Cognitive Assessment (MoCA)**

**NO:_______ Gender:_______ Age:__________**

**Education Background:____________**

| **Visual Space and Executive Function** | |
| --- | --- |
| **1.Alternate Connection Experiment**  戊  甲  乙  2  1  5  丁  丙  4  3  开始  结束 | **2. Replicating Cube** |
| **3.Draw a Clock at 11:10** | **4.Nominate** |
|  | 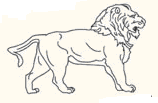  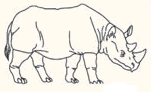  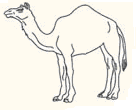 |

| **Attention** | **Repeat the following figures**  **(count one per second).** | | | | Count in order | | | **21854** |
| --- | --- | --- | --- | --- | --- | --- | --- | --- |
|  |  |  |  |  | Count backwards | | | **742** |
|  | **Every time when it appears 1, knock the desk once** | | | **52139411806215194511141905112** | | | | |
|  | **100 minus 7 in succession** | **93** | **86** | **79** | | **72** | **65** | |

| **Memorize** | Read the following terms,and then repeat the above process two times,finally recollect after five minutes. |  | **Face** | **Velvet** | **Church** | **Chrysanthemum** | **Redness** |
| --- | --- | --- | --- | --- | --- | --- | --- |
|  |  | **1st** |  |  |  |  |  |
|  |  | **2nd** |  |  |  |  |  |

| **language** | Repetition:  I just know that it is Zhang Liang who comes to help today.  When dog is at the room, cat always hide under the sofa. |
| --- | --- |
|  | Fluency: Speak animals as much as you can in one minute. ________(N≥11 animals) |

| **Abstraction** | Similarity in terms:   1. Banana-orange=fruit 2. Train-bicycle 3. Watch-ruler |
| --- | --- |

| **Postpone recollection** | **No tips while recollecting** | **Face** | **Velvet** | **Church** | **Chrysanthemum** | **Redness** |
| --- | --- | --- | --- | --- | --- | --- |
|  | **Tips in classification** |  |  |  |  |  |
|  | **Tips in multiple choices** |  |  |  |  |  |

| **Orientation** | **Date** | **Month** | **Year** | **Week** | **Site** | **City** |
| --- | --- | --- | --- | --- | --- | --- |
|  |  |  |  |  |  |  |
