# Supplementary material for: Long-term high physical activity modulates event-related potential indices of inhibitory control in postmenopausal women
Source: PeerJ. 2019 Mar 18;7:e6523. doi: 10.7717/peerj.6523 (PMC6428037; doi:10.7717/peerj.6523)
Supplement: Supplemental Information 4 [file peerj-07-6523-s004.docx]

**INTERNATIONAL PHYSICAL ACTIVITY QUESTIONNAIRE ( An Extract)**

**ID: Name:**

The questionnaire is designed to track your various physical activities in the past seven days, including the time you spent in your work, taking transportation, doing chores at home and leisure time(physical exercise and entertainment)

In the following questions,

The vigorous physical activities refers to the activities that take your great efforts to finish in at least ten minutes once time, while you breathe much harder than normal with your heart beating apparently faster.

The moderate physical activities refer to activities that takes your moderate efforts to finish in at least ten minutes once time, while you breathe quite normal or slightly hard with your heart beating faster a bit.

**Background information:**

Weight: ____kg Height:______cm Age:_____

The lightest time:__________

Profession before retiring:________ Still work at present?________________

**When you answer the following questions, please just take physical activities that occupy you at least ten minutes every time into consideration.**

1. In the past seven days, how many days have you engaged in vigorous physical activities, such as carry (lift) heavy stuff, ascend the stairs, climb mountains, shovel with a hoe, move furniture, ride bicycles fast, vigorous physical activities(running, rope skipping, or setting-up exercise in a gym)? (only the activities that taking at least 10 minutes are accounted)

_____day(s) per week. No any vigorous activities, please skip to question 3

1. During these days, how many hours have you spent in those vigorous activities?

Average ___hour(s)_____minute(s) per day

1. In the past seven days, how many days have you engaged in moderate physical activities, such as carry(lift) light stuff, clear up yards or balconies, sweep the floor with hands, wash clothes with hands, clean the doors and windows, trim flowers and grasses with a shear, make beds, soothe your baby by walk around, wash your car by hands, descend stairs, play badminton,play ping-pong, ride a bicycle at a normal speed(take it as a vehicle tool), join a dancing party, do Tai Chi or Mulan boxing? _______

**Note:** don’t take sweep the floor with broom, mop the floor, cut vegetables or wash clothes in washing machine into account.

_____day(s) per week. No any moderate activities, please skip to question 5

1. During these days, how many hours have you spent in those moderate activities?

Average ___hour(s)_____minute(s) per day

1. In the past seven days, how many days have you engaged in walking at least ten minutes every time? The walking here includes the steps done in your workplace, at your home, during the transportation journey or for the purpose of exercising.

_____day(s) per week. No walking continuously more than ten minutes, please skip to question 7.

1. During these days, how many hours have you spent in walking?

Average ___hour(s)_____minute(s) per day

1. In the past seven days, how long have you been in sedentariness every day? Including the time at home, in your workplace or your leisure time. It could happen to the time you spent in visiting friends or relatives, reading books or newspapers, sitting, or watching TV in sprawling. Please don’t count your sleeping time.

Average ___hour(s)_____minute(s) per day

**Thanks for your participation!**
